# Chitosan Tubes Enriched with Fresh Skeletal Muscle Fibers for Primary Nerve Repair

**DOI:** 10.1155/2018/9175248

**Published:** 2018-06-13

**Authors:** Giulia Ronchi, Benedetta Elena Fornasari, Alessandro Crosio, Claudia Alexandra Budau, Pierluigi Tos, Isabelle Perroteau, Bruno Battiston, Stefano Geuna, Stefania Raimondo, Giovanna Gambarotta

**Affiliations:** ^1^Department of Clinical and Biological Sciences, University of Torino, Torino, Italy; ^2^Neuroscience Institute Cavalieri Ottolenghi, University of Torino, Torino, Italy; ^3^Department of Orthopedics and Traumatology II, Surgery for Hand and Upper Limb, AOU Città della Salute e della Scienza di Torino, CTO Hospital, Torino, Italy; ^4^UO Microchirurgia e Chirurgia della Mano, Ospedale Gaetano Pini, Milano, Italy

## Abstract

Muscle-in-vein conduit is successfully employed for repairing nerve injuries: the vein prevents muscle fiber dispersion, while the muscle prevents the vein collapse and creates a favorable environment for Schwann cell migration and axon regrowth. However, it requires microsurgical skills. In this study we show a simple strategy to improve the performance of a chitosan hollow tube by the introduction of fresh skeletal muscle fibers. The hypothesis is to overcome the technical issue of the muscle-in-vein preparation and to take advantage of fiber muscle properties to create an easy and effective conduit for nerve regeneration. Rat median nerve gaps were repaired with chitosan tubes filled with skeletal muscle fibers (muscle-in-tube graft), hollow chitosan tubes, or autologous nerve grafts. Our results demonstrate that the fresh skeletal muscle inside the conduit is an endogenous source of soluble Neuregulin 1, a key factor for Schwann cell survival and dedifferentiation, absent in the hollow tube during the early phase of regeneration. However, nerve regeneration assessed at late time point was similar to that obtained with the hollow tube. To conclude, the muscle-in-tube graft is surgically easy to perform and we suggest that it might be a promising strategy to repair longer nerve gap or for secondary nerve repair, situations in which Schwann cell atrophy is a limiting factor for recovery.

## 1. Introduction

Currently, the gold standard technique used to repair large peripheral nerve defects is the autologous nerve graft. However, this procedure has some well-known disadvantages: the need of an additional surgery to harvest the donor nerve, sensory deficits at the donor site, the possibility of neuroma-in-continuity formation with consequent neuropathic pain, and the limited availability of donor nerves in terms of number and diameter [1–3]. These limitations inspired researchers to develop alternative techniques for repairing large nerve defects.

The use of conduits made by nonnervous materials (tubulization technique) to bridge nerve gap has been widely investigated and has shown promising results both experimentally and clinically [2, 4]. Tubular conduits have proven to be an excellent alternative to autologous nerve grafts because they act as physical guidance for the regenerating axons and provide a protective environment for axonal growth by limiting surrounding tissue invasion and by reducing neuroma and scar tissue formation [5]. Moreover, they facilitate the accumulation and the concentration of neurotrophic and neurotropic factors produced by the injured nerve stumps [6, 7].

Several materials, both of biological and of synthetic origin, have been used to build tubular conduits [8]. Among these, muscle-in-vein combined conduits have been extensively and effectively employed to repair injured nerves. The success of this technique is due to the presence of the vein that prevents muscle fiber dispersion and scar tissue invasion and muscle fibers which prevent the vein collapse and create a favorable environment for Schwann cell migration and axonal regrowth [9]. However, the muscle-in-vein graft is technically challenging and requires microsurgical skills.

Chitosan is a natural polysaccharide that has been demonstrated to be a good biomaterial with a wide range of biomedical and tissue engineering applications [10, 11] due to its biocompatibility, biodegradability, low toxicity, and bioactivity. In recent studies its regenerative properties have been demonstrated in both standard and critical length nerve reconstruction [10, 12, 13]. These promising preclinical results confirmed that chitosan is a suitable biomaterial for peripheral nerve regeneration and in 2014 chitosan tubes have been accepted for clinical use (Reaxon^®^ Nerve Guide, Medovent GmbH, Mainz, Germany).

Nevertheless, when more complex lesions occur, such as long gaps, hollow tubes not always lead to good results and conduit enrichment might create a favorable environment for nerve regrowth.

In this study we combined the use of a successful conduit (the chitosan tube) with the promising and simple intraluminal structure (fresh longitudinal skeletal muscle fibers) to evaluate peripheral nerve regeneration after rat median nerve reconstruction. The hypothesis is to (i) overcome the technical issue of the muscle-in-vein preparation by the use of a successful conduit, and to (ii) take advantage of fiber muscle properties to create a surgically easy and effective conduit for nerve regeneration.

Rat median nerve gaps were repaired with (i) chitosan tubes filled with skeletal muscle fibers (muscle-in-tube graft), (ii) hollow chitosan tubes, or (iii) autologous nerve grafts (surgical gold standard). Samples harvested at early (1, 7, 14, and 28 days after nerve repair) time points, together with* in vitro* analysis, demonstrated that fresh skeletal muscle produces and releases soluble NRG1, a key factor for Schwann cell survival and dedifferentiation present also in the autograft but absent in the hollow tube in the first phases of nerve regeneration. However, nerve regeneration assessed at a late time point (12 weeks) is comparable to that obtained with the hollow chitosan tube and, as shown in previous studies [14, 15], with the autograft, in terms of functional recovery, number and size of regenerating fibers.

## 2. Methods

### 2.1. Conditioned Medium for Cell Stimulation and ELISA

Longitudinal pieces of rat* pectoralis major* muscle about 1 cm long were put in culture immediately after harvesting in a 24-multiwell using 0.5 ml (1, 3 days) or 1 ml (7, 14 days) 2% FBS DMEM/piece. Supernatant was collected, spun at 3000 rpm at room temperature for 5 minutes, and immediately frozen in liquid nitrogen for the following ELISA analysis. To quantify soluble Neuregulin 1 (NRG1) in the muscle conditioned medium, ELISA analyses (#900-M316, PeproTech) were performed following manufacturer's instructions. 2% FBS DMEM was used as background negative control. To investigate ELISA specificity, recombinant NRG1*α* and NRG1*β*1 purchased from R&D (#296-HR-050 and #396-HB-050) were also analyzed.

At the same time points, degenerating muscles were collected and frozen for RNA extraction and quantitative real time PCR (qRT-PCR) analyses. As control for RNA analysis, fresh muscle fibers were also collected. Experiments were carried out in biological triplicate.

### 2.2. RNA Isolation, cDNA Preparation, and Quantitative Real Time PCR

Total RNA was isolated according to the manufacturer's instructions using TRIzol Reagent (Invitrogen). Reverse transcription and quantitative real time PCR were performed and analyzed as previously described [16]. 0.75 *μ*g of total RNA was retrotranscribed, the cDNA was diluted 10-fold, and 5 *μ*l (corresponding to 15ng of the starting RNA) as analyzed for each PCR reaction. The presence of a single peak corresponding to the required amplicon was verified and dissociation curves were routinely performed. Technical and biological triplicates were performed.

As calibrator for relative quantification, the average of uninjured nerve ΔCt was used for nerve regeneration* in vivo* experiments and the average of fresh muscle ΔCt was used for muscle degradation* in vitro* analysis.

As housekeeping gene to normalize data, ANKRD27 (Ankyrin repeat domain 27) was used for* in vivo* nerve analysis and TBP (TATA Binding protein) was used for* in vitro* muscle analysis. Primer sequences for ErbB1, ErbB2, ErbB3, NRG1 type I/II, type III, *α*, *β*, type a, type b, type c, and ANKRD27 were previously published [16]. New unpublished primer sequences were prepared for ErbB4 (accession number #AY375307.1, amplicon length 103 bp): ErbB4 forward: 5' AAGTTCTGGATGCGGAAGATGCC-3' and ErbB4 reverse: 5'-TTGTTCAGCACACACAGTCCTGG-3'. For NRG1*α* e *β* the forward primer is common: 5'-CGACTGGGACCAGCCATCTCATAAAG-3'; NRG1*α* reverse: TTGCTCCAGTGAATCCAGGTTG (accession number: U02324, amplicon length: 116 bp); NRG1*β* reverse: 5'-AACGATCACCAGTAAACTCATTTGG-3' (accession number: U02322, amplicon length: 144 bp). Melting temperature for all primer pairs was set at 60°C.

### 2.3. Surgery

For* in vivo* study 68 female adult Wistar rats were used (Harlan, weight: 200-250g). Animals were housed in a room with controlled temperature and humidity, with 12h of light and 12h of dark and free access to food and water. Every attempt was made to reduce animal suffering. All procedures were approved by the Bioethical Committee of the University of Torino and by the Italian Ministry of Health. Moreover, these procedures agree with the National Institutes of Health guidelines, the Italian Law for Care and Use of Experimental Animals (DL26/14), and the European Communities Council Directive (2010/63/EU).

**Figure 1:**
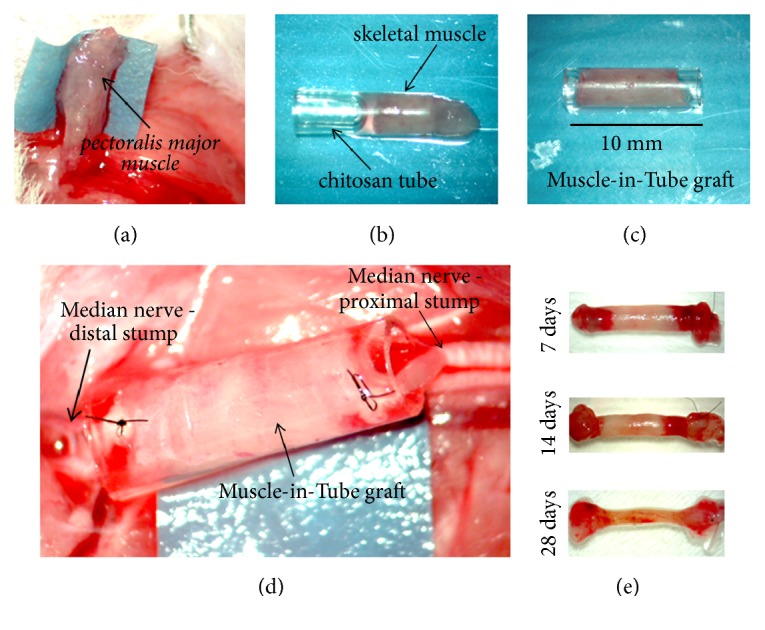
Preparation of the muscle-in-tube graft. A longitudinal piece of the* pectoralis major* muscle was removed (a) and used to fill the chitosan conduit (b), to obtain a 10 mm long muscle-in-tube graft (c). The graft was then used to repair a median nerve defect (d). Pictures showing the regenerated nerves at different time points postsurgery (e). The conduit has been removed and a suture was used to mark the proximal stump (on the right in the pictures).

During surgical procedures, animals were placed under general anaesthesia induced by IM injection of Tiletamine + Zolazepam (Zoletil, 3 mg/kg) and were positioned in supine position. Using an incision from the nipple to the elbow, the median nerve was isolated to establish a defect in the middle of the exposed part, immediately followed by nerve repair according to the experimental group:

(i)* Chitosan-based hollow tube*: a nerve segment was removed and a 10 mm long chitosan tube was used to bridge the nerve defect by inserting 1 mm of the two nerve ends inside the conduit. The nerve guide was sutured at each end.

(ii)* Chitosan-based tube filled with fresh skeletal muscle—“ muscle-in-tube graft”*: a nerve segment was removed and a 10 mm long chitosan tube enriched with a longitudinal piece of the* pectoralis major* muscle (withdrawn from the same animal) was used to bridge the nerve defect by inserting 1 mm of the two nerve ends inside the conduit. The nerve guide was sutured at each end, as shown in Figure 1.

(iii)* Autograft*: a median nerve segment was cut out, reversed (distal-proximal), and sutured to the nerve ends of the same animal. Chitosan-based tubes (Reaxon Nerve Guide) were supplied by Medovent GmbH, Germany.

Animals were sacrificed by anaesthetic overdose at different times points: 1 day (only autograft and muscle-in-tube graft) and 7, 14, and 28 days for early time point analysis; 1 day hollow chitosan tube graft was not analyzed, because it is colonized only by fluid and it was not possible to withdraw it. For late time point analysis (only hollow tube and muscle-in-tube), animals were sacrificed 12 weeks after the surgery. Data about autograft 12 weeks after surgery are already present in literature [14, 15]. For each time point (1, 7, 14, and 28 days) n=4 samples for each experimental group were harvested for biomolecular analysis and n=1 for qualitative morphological analysis (7, 14, and 28 days). For morphometrical analysis, 12 weeks regenerated distal stumps were harvested (n=5).

Healthy median nerve segments and healthy* pectoralis major* muscles from other rats were also harvested as control in the qRT-PCR analysis.

### 2.4. Grasping Test

The grasping test was performed to estimate the functional recovery after nerve reconstruction. The analysis was carried out before animal sacrifice (12 weeks after nerve repair) following the same procedure previously described [17]. Rats were acclimatize before the testing. Each animal was tested three times and the average value was recorded.

### 2.5. Resin Embedding, High Resolution Light Microscopy, and Electron Microscopy Analysis

Samples corresponding to the grafted region (the autologous nerve and the filler of the chitosan tubes) harvested 7, 14, and 28 days after repair and the distal portion of regenerated median nerve, harvested 12 weeks after repair, were processed for resin embedding as previously described and semithin and ultrathin sections were cut for light and electron microscopy analysis [14].

### 2.6. Morphometrical Analysis

The quantification of myelinated nerve fibers was performed on electron microscopy micrographs. Briefly, on one randomly selected ultrathin section, 15-20 fields were chosen using a systematic random sampling protocol, as earlier described [18], with a magnification of 4000X. In each sampling field, a two-dimensional (2D) dissector procedure was used [18]. Mean fiber density, total fiber number, fiber and axon diameter, myelin thickness, and g-*ratio* were then estimated. The total cross-sectional area of the whole nerve was measured on the toluidine-blue-stained semithin section.

### 2.7. Statistical Methods

For statistical analysis IBM SPSS Statistics 22.0 software was used. Data were expressed as mean ± standard error of the mean (SEM). Data sets containing two groups were analyzed through two-tailed Student's* t*-test, while data sets with more than two groups were processed with one-way analysis of variance (ANOVA) with Bonferroni correction to compare at the different time points the three experimental models of nerve repair and with two-way ANOVA to analyze the influence of the two factors (experimental model of nerve repair and time after repair).

## 3. Results

### 3.1. In Vitro Analysis: Soluble Neuregulin 1 Is Expressed and Released by in Vitro Degenerating Muscles

**Figure 2:**
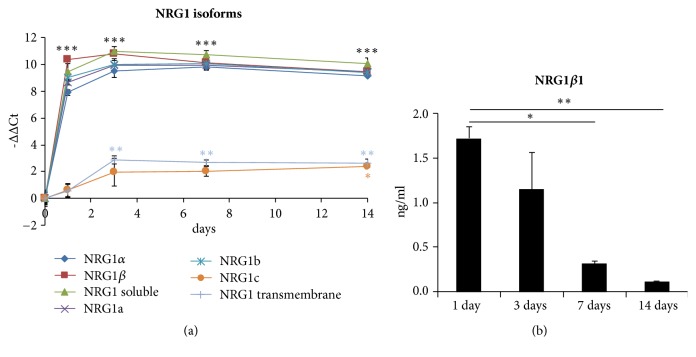
Degenerating muscle expresses NRG1. (a) qRT-PCR showing the relative quantification (-ΔΔCt = log_2_fold change) of the different NRG1 isoforms in* in vitro* degenerating muscle. TBP was used as housekeeping gene to normalize data. (b) ELISA data showing NRG1*β*1 release in the medium of degenerating muscles. Data are expressed as mean ± SEM. Asterisks denote significant differences between degenerating muscles and the healthy muscle (a) or between the released NRG1 amounts at the different time points (b), ^*∗*^p ≤ 0.05, ^*∗∗*^p ≤ 0.01, and ^*∗∗∗*^p ≤ 0.001.

To evaluate the expression level of the different Neuregulin 1 (NRG1) isoforms in degenerating muscles, longitudinal pieces of the* pectoralis major* muscle were put in culture in 0.5 ml 2% FBS medium immediately after harvesting. At different time points (1, 3, 7, and 14 days) total RNA was extracted from* in vitro* degenerating muscles and from control healthy muscles and the expression level of different NRG1 isoforms was analyzed by quantitative real time PCR, discriminating among soluble and transmembrane, *α* and *β*, type a, type b, and type c isoforms. Data analysis showed that, during* in vitro* degradation, soluble isoforms were strongly and significantly upregulated and that soluble upregulated isoforms belonged to *α* and *β*, type a, and type b isoforms; transmembrane and type c isoforms were also upregulated, although with a high threshold cycle (Ct) indicative of a low expression level (Figure 2(a)).

Conditioned medium was collected and analyzed by ELISA to quantify soluble NRG1 release. ELISA was performed on 50 *μ*l muscle conditioned medium collected after 1, 3, 7, and 14 days of* in vitro* culture. To investigate if ELISA antibodies were able to recognize both NRG1*α* and NRG1*β*, additional standard curves were performed using recombinant proteins; nevertheless, only NRG1*β* isoforms were recognized (data not shown). ELISA data analysis showed that muscle was able to release soluble NRG1*β* in the medium (1.721 ng/ml ± 0.231 after 24 hours, Figure 2(b)).

### 3.2. *In Vivo* Analysis

To verify the effectiveness of the muscle-in-tube graft to repair rat median nerve gaps, we compared this method with hollow chitosan tubes and autologous nerve graft.

#### 3.2.1. Short-Term Analysis of Regenerated Nerve: Qualitative Morphology

**Figure 3:**
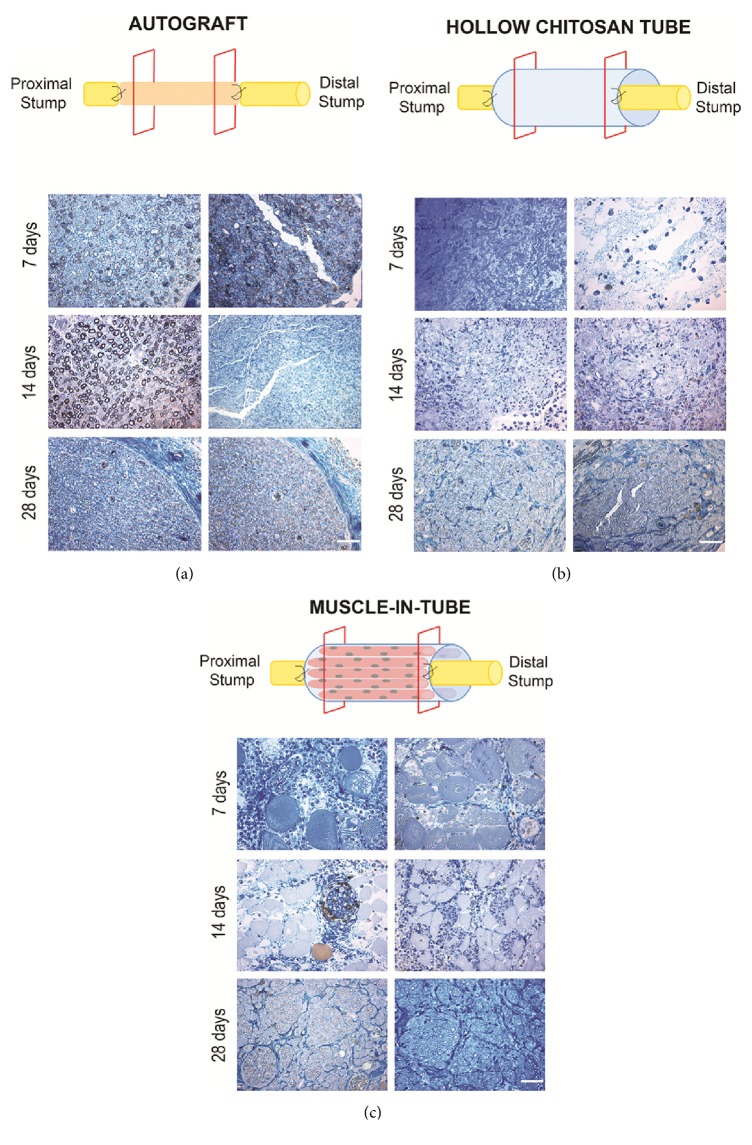
Representative high resolution light images of toluidine-blue stained semithin transverse sections of regenerated median nerve repaired with autograft (a), hollow chitosan tube (b), or muscle-in-tube graft (c). Images were taken inside the grafts, both proximally (left columns, about 1,5 mm from the proximal suture point) and distally (right columns, about 1,5 mm from the distal suture point), at different time points (7, 14, 28 days after repair). Bar: 40 *μ*m.

To observe the qualitative morphology of the nerve in the early stages of regeneration, high resolution light micrographs of toluidine-blue stained semithin sections were taken at short-term time points postsurgery (7, 14 and 28 days). Images were gathered inside the different grafts, both proximally and distally, to follow nerve regeneration alongside the conduits (Figure 3).

Nerves repaired with the autograft (positive control) showed an early regeneration, as expected (Figure 3(a)). 7 days after implantation, the nerve fibers inside the graft underwent Wallerian degeneration and the graft was filled with axons and myelin debris. After 14 days regenerated fibers were already present in the proximal part of the graft, while in the distal part endoneurial tubes were ready to be colonized by regrowing axons. Finally, after 28 days the whole graft was colonized by regenerated fibers.

Regeneration inside the hollow chitosan tube is slower when compared to the autograft (Figure 3(b)). Few days after implantation, extracellular matrix and fluid containing inflammatory cells filled the conduit. After 14 days, several cell types colonize the conduit and only after 28 days were the myelinated fibers detectable inside the conduit. Regenerated fibers were compacted in the central part of the tube (Figure 3(b)).

In the muscle-in-tube graft, longitudinal muscle fibers were inserted inside the chitosan conduit to provide a physical and trophic scaffold for axon and cell growth (Figure 3(c)). 7 days after nerve repair, big muscle fibers were clearly detectable inside the graft, together with different cell types. After 14 days, muscle fibers appeared smaller, showing that they were undergoing progressive degeneration over time. Few myelinated fibers organized in small fascicles were detectable. Finally, 28 days after implantation, the conduit was colonized by a high number of myelinated fibers organized in fascicles. Among nerve fascicles, small degenerating muscle fibers were still visible.

#### 3.2.2. Short-Term Analysis of Regenerated Nerve: Biomolecular Evaluation

**Figure 4:**
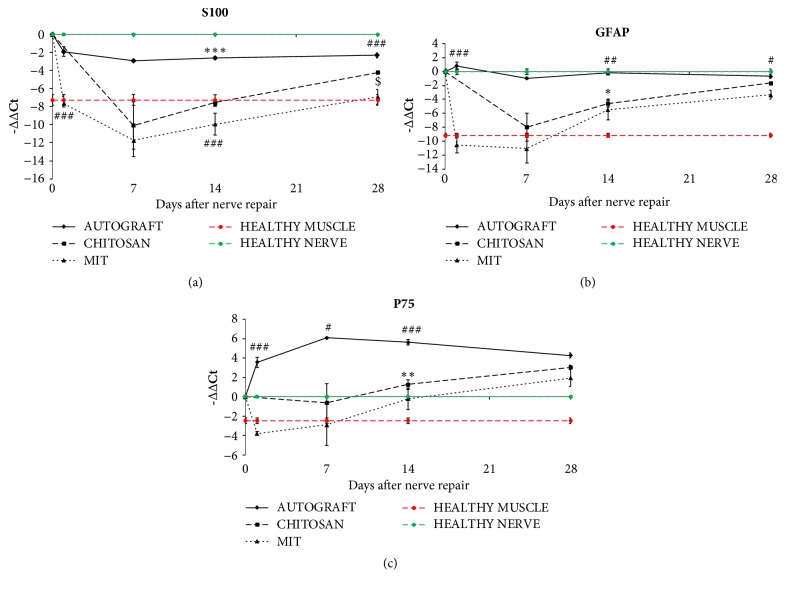
Quantitative analysis of SC marker expression. Relative quantification (-ΔΔCt = log_2_fold change) of S100 (a), GFAP (b), and p75 (c) was evaluated by qRT-PCR. Both healthy nerves and healthy muscle fibers were analyzed and shown in the figures (green and red lines, respectively). ANKRD27 was used as housekeeping gene to normalize data. Values in the graphics are expressed as mean ± SEM. One-way ANOVA was carried out; asterisks (*∗*) denote statistically significant differences between autograft and hollow chitosan tube groups; hashes (#) between autograft and muscle-in-tube groups; dollars ($) between hollow chitosan tube and muscle-in-tube graft. ^*∗*/#/$^p ≤ 0.05, ^*∗∗*/##/$$^p ≤ 0.01, and ^*∗∗∗*/###/$$$^p ≤ 0.001. Two-way ANOVA is shown in Table S1.

**Figure 5:**
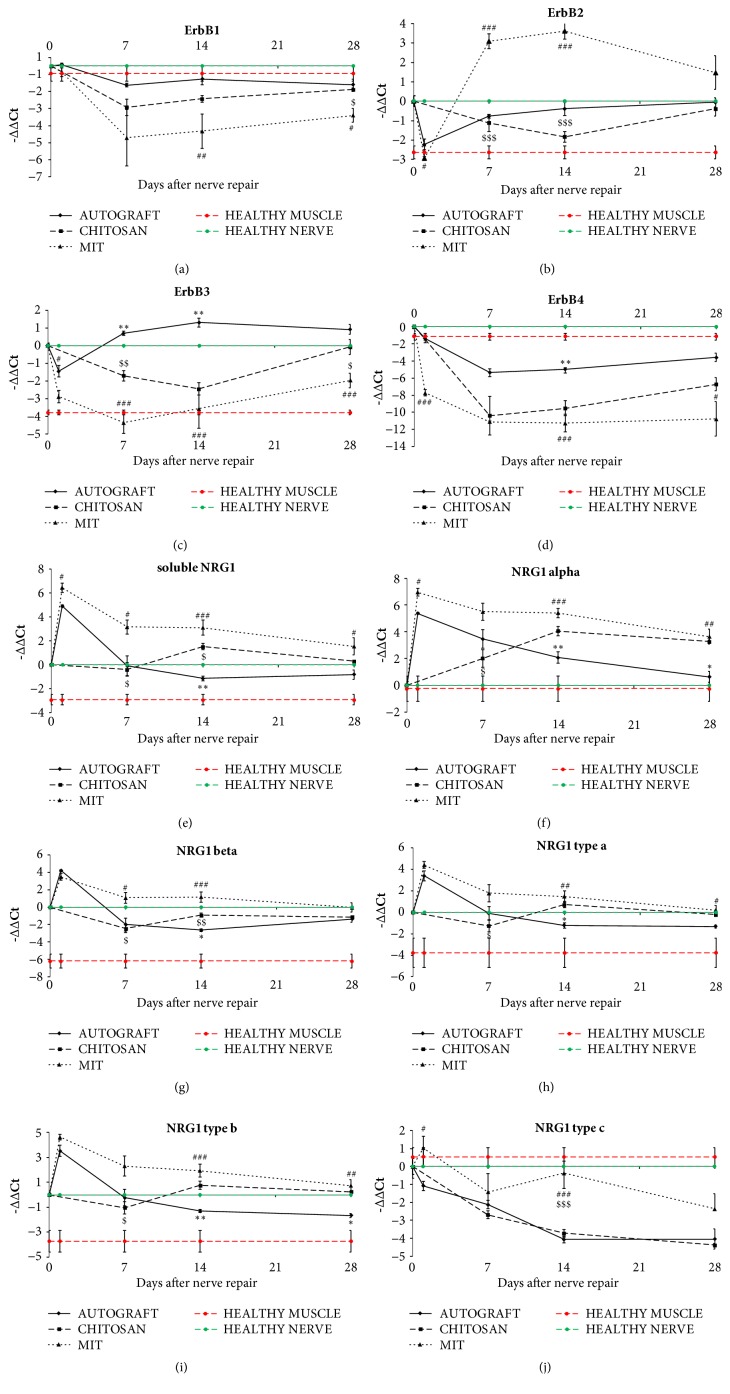
Quantitative analysis of the NRG1/ErbB system expression. qRT-PCR showing the relative quantification (-ΔΔCt = log_2_fold change) of ErbB receptors (a-d) and the different NRG1 isoforms (e-j). Both healthy nerves and healthy muscle fibers were analyzed and shown in the figures (green and red lines, respectively). ANKRD27 was used as housekeeping gene to normalize data. Values in the graphics are expressed as mean ± SEM. One-way ANOVA was carried out; asterisks (*∗*) denote statistically significant differences between autograft and hollow chitosan tube groups; hashes (#) between autograft and muscle-in-tube groups; dollars ($) between hollow chitosan tube and muscle-in-tube graft.  ^*∗*/#/$^p ≤ 0.05, ^*∗∗*/##/$$^p ≤ 0.01, and ^*∗∗∗*/###/$$$^p ≤ 0.001. Two-way ANOVA is shown in Table S1.

At different time points after nerve reconstruction (1, 7, 14, and 28 days), three Schwann cell markers (S100, p75, GFAP), ErbB receptors, and different NRG1 isoforms were examined by qRT-PCR in the grafts of the three experimental groups (Figures 4 and 5). For the hollow tube, we analyzed the samples starting from day 7 after repair, because 1 day after repair the tube was colonized only by fluid material and was not possible to withdraw and analyze it. Because the hollow chitosan tube and the muscle-in-tube graft at time zero do not contain Schwann cells, the starting point of expression analysis should be considered 7 days for the hollow chitosan tube and 1 day for muscle-in-tube graft.

The expression of Schwann cell markers S100 and GFAP was significantly lower in hollow chitosan tube and muscle-in-tube compared to both the autograft and the healthy nerves. After 28 days muscle-in-tube graft still showed a lower expression of these two markers (Figures 4(a) and 4(b)).

p75 expression was significantly higher in the autograft samples at the different time points, while after 28 days no differences among the three repair experimental models were detectable (Figure 4(c)).

Then, ErbB receptors and NRG1 isoforms were analyzed. ErbB1 expression level in the muscle-in-tube samples was downregulated after injury and repair and was lower than in the hollow tube and in the autograft samples, where the expression was similar (Figure 5(a)).

ErbB2 expression in the muscle-in-tube samples was strongly and stably upregulated at 7 and 14 days after injury relatively both to the healthy nerve and muscle and to the autograft and the hollow chitosan groups. At day 14 the autograft showed an expression level higher than the empty chitosan group (Figure 5(b)).

ErbB3 expression in the chitosan tube and in the muscle-in-tube after injury was lower than in the autograft samples. 28 days after injury ErbB3 expression in the hollow chitosan samples was similar to the autograft and higher than the muscle-in-tube samples (Figure 5(c)).

ErbB4 expression is significantly lower in the muscle-in-tube samples—relatively to both healthy muscle and nerve—already 1 day after injury and remained really low until 28 days. In the hollow chitosan samples ErbB4 expression was similar to the muscle-in-tube samples, while in the autograft ErbB4 expression decreased after injury but was higher than the muscle-in-tube graft and the hollow chitosan samples. 28 days after injury only hollow chitosan samples showed an expression level similar to the autograft (Figure 5(d)).

NRG1 expression analysis is really complex, because each primer pair amplifies a single isoform, but soluble and transmembrane isoforms can be type *α* or type *β*, and type a, b, or c. Transmembrane NRG1 expression was also analyzed but not shown, because its expression level is barely detectable, being this isoform expressed mainly by the axon and not by Schwann cells.

Autograft showed a strong early upregulation (1 day) of the different NRG1 isoforms (except for NRG1c) followed by a return to values similar to control nerves. On the contrary, NRG1c was downregulated from the 14th day (Figures 5(e)–5(j)).

In the first days after implantation, the hollow chitosan tube is filled by cells which do not express NRG1 or that express it at low level; indeed, at day 7 mRNA expression of most NRG1 isoforms was similar or lower to control nerve values. At day 14 NRG1 *α* started to be overexpressed and was still upregulated after 28 days (Figures 5(e)–5(j)).

Finally, muscle-in-tube graft showed an expression pattern very similar to that of the autograft: all soluble NRG1 isoforms showed a strong peak 1 day after implantation (except for NRG1c) and then the upregulation of most of them was still high at 7 and 14 days, returning to control values after 28 days (except for NRG1 *α*, that was still upregulated also after 28 days) (Figures 5(e)–5(j)).

Overall, the upregulation peak of NRG1 isoforms in the hollow chitosan tube started only 14 days after injury and is lower than muscle-in-tube graft. Intriguingly, the upregulation at day 1 of soluble NRG1 (*α* and c isoforms) in muscle-in-tube samples was stronger than in autograft samples.

Two-way ANOVA was carried out for all analyzed genes to evaluate the effect on their expression of the different repair experimental models and of the time after repair (see Figure S1). For all genes, statistical analysis with two-way ANOVA revealed significant main effects of the repair experimental model (dF=2, P<0.0001) and of the time (dF=4, P<0.0005); interaction between the 2 factors was revealed to be statistically significant (dF=7, P<0.05) for all genes except ErbB4 (P=0,056) and NRG1c (P=0.053).

#### 3.2.3. Long-Term Analysis of Regenerated Nerve: Functional, Morphological, and Morphometrical Analyses

**Figure 6:**
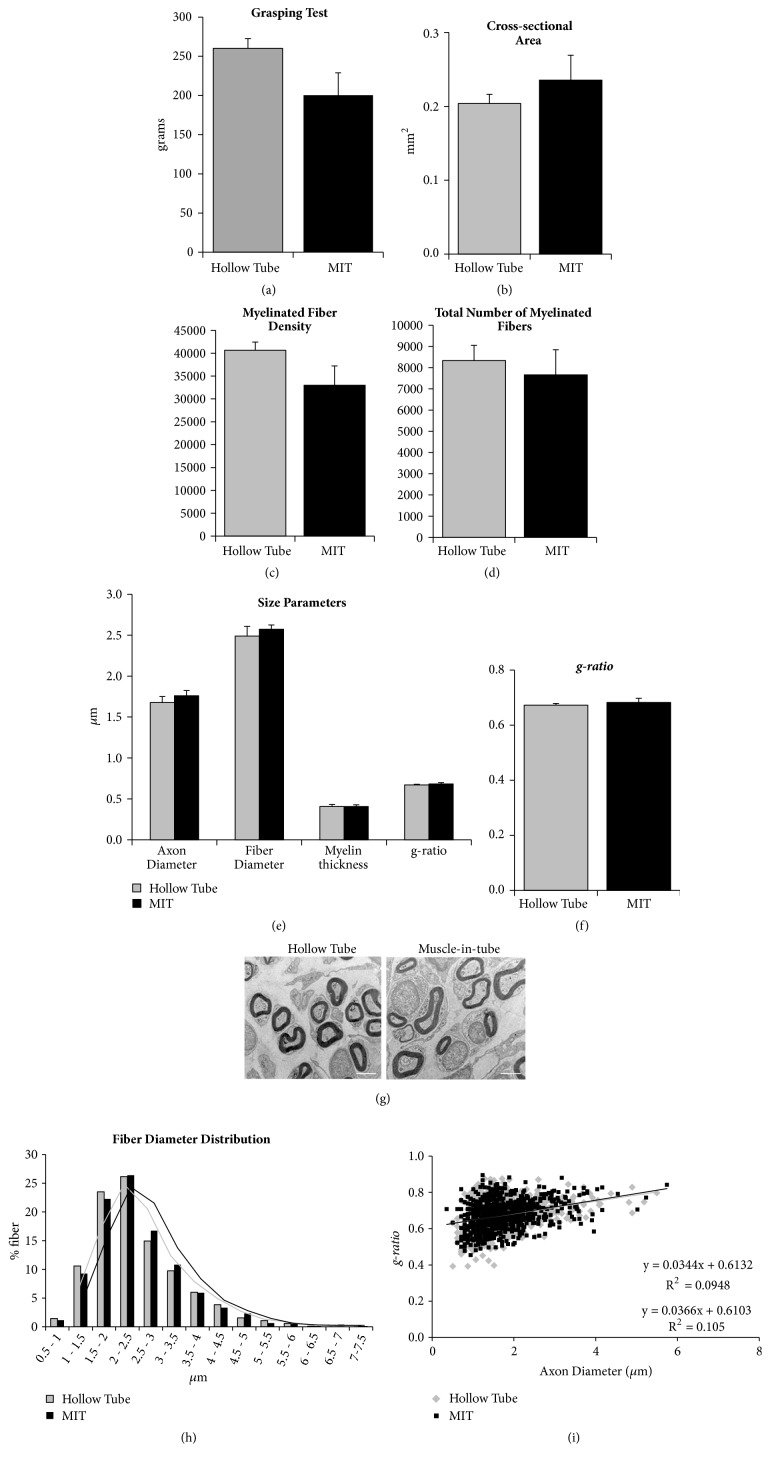
Functional, morphological, and morphometrical analyses of long-term regenerated nerves after hollow tube or muscle-in-tube (MIT) graft primary repair. (a) Histograms showing the result of the grasping test, performed 12 weeks after nerve repair. (b-f) Histograms of morphometrical analysis of the regenerated myelinated fibers. Analyses were performed 3 mm distal to the graft: (b) cross-sectional area of the whole nerve section, (c) myelinated fiber density, (d) total number of myelinated fibers, (e) size parameters (axon and fiber diameter, myelin thickness), and (f) g*-ratio*. Values in the graphics are expressed as mean + SEM. (g) Representative electron microscopy images of the regenerated distal part of the two experimental groups. Bar: 2 *μ*m. (h) Frequency distribution histograms of myelinated fiber diameters. The two lines are the fitting lines of the distribution. (i) Scatter plots showing g*-ratio* of individual myelinated axons as a function of axon diameter. Regression lines, their equations, and R^2^ are also shown. Both (h) and (i) data were obtained by pooling all the values from all animals for each experimental group; hollow tube: n=830; muscle-in-tube graft: n=777.

To determine whether the presence of fresh muscle fibers inside the chitosan tube influences nerve regeneration, we performed a functional test (grasping test) and morphometrical analysis on the nerve segment distal to the graft 12 weeks after nerve reconstruction (Figure 6). Analyses were performed at electron microscopy level to enable accurate identification of all myelinated nerve fibers.

The functional test performed at week 12 after repair did not show statistical differences between the hollow chitosan tube and the muscle-in-tube groups (Figure 6(a)).

Results of the quantitative analysis performed on regenerated nerve fibers show no significant differences in all the analyzed parameters between the two experimental groups (Figures 6(b)–6(f)), as showed also by representative images (Figure 6(g)). The frequency distribution of nerve fiber diameters showed a shift of the muscle-in-tube histograms towards bigger size (Figure 6(h)). Finally, scatter plots displaying g-*ratios* of individual fibers in relation to respective axon diameter showed no differences between groups (Figure 6(i)).

## 4. Discussion

The use of hollow conduits to repair nerve defects is a valid alternative technique to autograft because of their well-demonstrated advantages, and in the last years several kinds of conduits have been proposed for their use in clinic.

However, the efficiency of hollow conduits is still insufficient, especially for large nerve gap, probably due to an inadequate formation of the extracellular matrix, thus limiting cell migration and axonal regrowth [6]. A number of intraluminal fillers of both biological and synthetic nature have been proposed to enrich hollow conduits [19]. Skeletal muscle fibers have been demonstrated to be a suitable intraluminal filler for several reasons. Firstly, the three-dimensional (3D) environment provided by the muscle basal lamina acts as a useful scaffold for growing axons and for migrating cells (Schwann cells, fibroblasts, and endothelial cells) [7]. Second, the muscle tissue is readily available at the nerve injury site and can be easily translated into clinical practice.

In 1993, Brunelli et al. [20] described an innovative biological conduit made by a vein segment filled with fresh skeletal muscle fibers (muscle-in-vein combined conduit). This technique result was experimentally successful and was therefore translated also to the clinic to treat certain cases of peripheral nerve injuries [21–23]. Nevertheless, the muscle-in-vein graft is not easy to perform and only few surgeons possess the technical skills necessary to build it.

To overcome this technical issue, the muscle tissue can be introduced inside a tubular conduit. However, so far, the placement of denatured skeletal muscle tissue [24–29] or fresh skeletal muscle fibers [30, 31] inside different kinds of tubular conduits (collagen, poly L-lactic acid and ∈-caprolactone–PLAC, or DL-lactide and ∈-caprolactone [p(DLLA-CL)]) has shown contrasting results in terms of nerve regeneration and functional recovery.

In this study we used a conduit made of chitosan (Reaxon Nerve Guide), that has been shown to promote successful nerve regeneration [12], combined with fresh longitudinal skeletal muscle fibers. The hypothesis is (i) to overcome the technical issue of the muscle-in-vein preparation and (ii) to take advantage of fiber muscle properties to create a surgically easy and effective conduit for nerve regeneration.

We used the fresh muscle tissue because it has been demonstrated that muscle cells produce and release factors that contribute to the survival of motoneurons* in vitro* [32]. Among released factors there are brain-derived neurotrophic factor, bone morphogenetic protein 6, cardiotrophin 1, glial cell-derived neurotrophic factor, heparan sulfate, hepatocyte growth factor, insulin-like growth factors, neurotrophin 3, neurotrophin 4, and vascular endothelial growth factor [32].

Here we focused our attention on Neuregulin 1 (NRG1), because it is known to be one of the most important factors regulating Schwann cell activity (survival, proliferation, dedifferentiation, and migration) and, therefore, it is a key factor for peripheral nerve regeneration.* In vitro* experiments performed in this study demonstrated that (i) skeletal muscle fibers upregulate the expression of soluble NRG1 mRNA while they are degenerating and that (ii) the soluble NRG1 protein produced by the muscle is released in the medium. Our results are in accordance with previous studies, showing a significant upregulation of soluble NRG1 after skeletal muscle denervation [33] and an increase in NRG1 mRNA in the muscle-in-vein combined grafts at early regeneration stages, while muscle is degenerating [34]. However, as far as we know, this is the first study showing the release of soluble NRG1 protein in the environment.

Taken together, these results show that the fresh skeletal muscle can be an endogenous source of the gliotrophic factor NRG1. For this reason, we moved to* in vivo* experiments using the rat median nerve experimental model for a primary repair of short gaps. The nerve gap has been repaired with the chitosan nerve guide (Reaxon Nerve Guide), filled with fresh skeletal muscle fibers (muscle-in-tube graft), and compared with the hollow one.

Our* in vivo* experiments aimed to (i) evaluate the different behavior of regenerating fibers inside the conduits (muscle-in-tube versus hollow tube) in terms of gene expression and axonal regrowth in the early stages of nerve regeneration (up to 28 days) and to (ii) investigate whether the conduit filled with fresh skeletal muscle fibers could improve nerve regeneration at later time points in terms of functional recovery and regeneration degree (12 weeks).

Our results indicate that muscle-in-tube graft promotes nerve regeneration. With respect to functional recovery and quantitative morphometry, no significant differences were observed between the two experimental groups at 12 weeks after surgery, suggesting that both conduits are effective for repairing peripheral nerve defects in this experimental model (short gap primary repair). The comparison of morphometrical data obtained in this study with our previous data obtained using autograft [14] shows that the regenerated fiber number is slightly lower in the two experimental groups compared to autograft group, but the size parameters are very similar. Also, functional recovery reached similar values between the two chitosan experimental groups and the autograft [15], confirming the efficiency of the muscle-in-tube graft in promoting nerve regeneration.

The mRNA analysis of these samples is really complex. Indeed, muscle-in-tube samples at early time points (1 day and 7 days after injury and repair) are in fact mostly muscles and for this reason we have to compare them also with the muscle, not only with the nerve. Then, step by step, the muscle mRNA percentage decreases, while the nerve mRNA percentage increases. Consequently, although healthy nerves were used as a common calibrator for the three experimental groups, both healthy nerves and healthy muscle fibers were analyzed and shown in the figures. Also hollow chitosan tubes represent a complex model, although in a different way: they need to be filled by migrating cell populations and for this reason the possible gene downregulation in the first time point (7 days) is only apparent, because at time zero they are just empty tubes. Therefore, 7 days should be considered the expression starting point for these samples.

mRNA analysis, performed inside the conduits at shorter time points, demonstrates that Schwann cell markers S100, p75, and GFAP are less expressed both in the hollow chitosan tube and in the muscle-in-tube graft when compared with the autograft. This is an expected result because the chitosan tube (empty or filled with muscle fibers) needs to be colonized by Schwann cells, whereas the autograft already contains Schwann cells that start to dedifferentiate immediately after repair. The slight (and insignificant) different expression between hollow chitosan tube and muscle-in-tube graft might be explained by the fact that in the muscle-in-tube graft the nerve RNA is “diluted” by the presence of muscle RNA.

Interestingly, hollow chitosan tube and muscle-in-tube graft differed greatly in terms of NRG1 expression: different isoforms of soluble NRG1 are highly expressed in the muscle-in-tube early after nerve repair, whereas no NRG1 expression is seen in the hollow chitosan tube. The NRG1 expression pattern observed in muscle-in-tube samples is very similar to that observed in autograft samples, in which NRG1 is upregulated by Schwann cells that colonize the graft. A similar expression pattern has also been described in other experimental models, such as crush injury and end-to-end repair [16], suggesting that soluble NRG1 isoforms are involved in the response to nerve injury, stimulating Schwann cell survival and promoting axon regrowth.

## 5. Conclusions

Our results show that the muscle-in-tube graft promotes nerve regeneration as efficiently as the hollow chitosan tube and that the fresh skeletal muscle inserted inside the chitosan conduit may be an endogenous source of soluble NRG1, a source that is absent in the hollow tube. We recently showed that, after prolonged degeneration of the median nerve distal stump, Schwann cells undergo atrophy and downregulate the expression of soluble NRG1. Even after the cross-suture with the freshly axotomized ulnar nerve proximal stump, NRG1 remains at very low expression level and nerve regeneration results are impaired [35]. Therefore, we suggest that soluble NRG1 supplied by fresh skeletal muscle-enriched conduit might be more useful to improve nerve regeneration in advanced experimental models, such as a longer gap or delayed (secondary) nerve repair.

## Data Availability

The raw data will be provided upon request.

## Supplementary Materials

Supplementary Materials
** Figure S1: **For quantitative real time PCR data, the effect of repair (autograft* versus* chitosan* versus* muscle-in-tube graft), the effect of the time after injury and repair (1* versus* 7* versus* 14* versus* 28 days), and the interaction between the two factors (repair *∗* time) were analyzed by two-way ANOVA test. Analysis of all genes showed highly significant main effects of repair and time. The interaction between the two factors is highly significant for most genes (dF = degrees of freedom, P = P value).
