# Supplementary material for: Chitosan Tubes Enriched with Fresh Skeletal Muscle Fibers for Primary Nerve Repair
Source: Biomed Res Int. 2018 Jun 13;2018:9175248. doi: 10.1155/2018/9175248 (PMC6020668; doi:10.1155/2018/9175248)
Supplement: Supplementary Materials — Figure S1: For quantitative real time PCR data, the effect of repair (autograft versus chitosan versus muscle-in-tube graft), the effect of the time after injury and repair (1 versus 7 versus 14 versus 28 days), and the interaction between the two factors (repair ∗ time) were analyzed by two-way ANOVA test. Analysis of all genes showed highly significant main effects of repair and time. The interaction between the two factors is highly significant for most genes (dF = degrees of freedom, P = P value). [file 9175248.f1.pdf]

factor	repair			time			repair * time		
	F	dF	P	F	dF	P	F	dF	P
ErbB1	15.254	2	0.000	16.671	4	0.000	2.49	7	0.033
ErbB2	39.186	2	0.000	28.479	4	0.000	9.121	7	0.000
ErbB3	65.198	2	0.000	16.261	4	0.000	9.942	7	0.000
ErbB4	32.770	2	0.000	50.926	4	0.000	5.568	7	0.056
soluble NRG1	22.407	2	0.000	50.770	4	0.000	8.517	7	0.000
NRG1 a	18.345	2	0.000	41.473	4	0.000	5.230	7	0.000
NRG1 b	22.407	2	0.000	50.770	4	0.000	8.51	7	0.000
NRG1 c	14.513	2	0.000	22.873	4	0.000	2.226	7	0.053
S100	32.807	2	0.000	24.346	4	0.000	3.214	7	0.010
p75	35.778	2	0.000	6.861	4	0.000	4.597	7	0.001
GFAP	26.718	2	0.000	11.616	4	0.000	4.154	7	0.002

Figure S1

For quantitative real time PCR data, the effect of repair (autograft *versus* chitosan *versus* muscle-in-tube), the effect of the time after injury and repair (1 *versus* 7 *versus* 14 *versus* 28 days) and the interaction between the two factors (repair * time) were analyzed by two-way ANOVA test. Analysis of all genes showed highly significant main effects of repair and time. The interaction between the two factors is highly significant for most genes (dF = degrees of freedom, P = P-value).
